# Pregnancy outcome following gestational exposure to azithromycin

**DOI:** 10.1186/1471-2393-6-18

**Published:** 2006-05-30

**Authors:** Moumita Sarkar, Cindy Woodland C, Gideon Koren, Adrienne RN Einarson

**Affiliations:** 1 The Motherisk Program, The Hospital for Sick Children, Toronto, Canada; 2 Department of Pharmaceutical Sciences, The University of Toronto, Canada; 3 Department of Pharmacology and Toxicology, The University of Toronto, Canada

## Abstract

**Background:**

Azithromycin is an azalide antibiotic with an extensive range of indications and has become a common treatment option due to its convenient dosing regimen and therapeutic advantages. Human studies addressing gestational use of azithromycin have primarily focused on antibiotic efficacy rather than fetal safety. Our primary objective was to evaluate the possibility of teratogenic risk following gestational exposure to azithromycin.

**Methods:**

There were 3 groups of pregnant women enrolled in our study: 1) women who took azithromycin. 2) women exposed to non-teratogenic antibiotics for similar indications, and 3) women exposed to non-teratogenic agents. They were matched for gestational age at time of call, maternal age, cigarette and alcohol consumption. Rates of major malformations and other endpoints of interest were compared among the three groups.

**Results:**

Pregnancy outcome of 123 women in each group was ascertained. There were no statistically significant differences among the three groups in the rates of major malformations; 3.4% (exposed) versus 2.3% (disease matched) and 3.4% (non teratogen) or any other endpoints that were examined. In the azithromycin group, 88 (71.6%) women took the drug during the first trimester

**Conclusion:**

Results suggest that gestational exposure to azithromycin is not associated with an increase in the rate of major malformations above the baseline of 1–3%. Our data adds to previous research showing that macrolide antibiotics, as a group, are generally safe in pregnancy and provides an evidence-based option for health professionals caring for populations with *chlamydia*.

## Background

Azithromycin (Zithromax^®^) is an azalide antibiotic belonging to a subclass of macrolide antibiotics developed as an alternative treatment to improve the spectrum of activity and to eliminate some of the clinical disadvantages associated with other macrolides [1]. Structural modification resulting from substitution of a nitrogen atom on the lactone ring with a methyl group, protects azithromycin from breakdown by gastric acidity and thereby increases its elimination half-life [2], making it possible for single dose therapy. Its unique pharmacokinetic properties offer additional clinical advantages that include a substantial reduction of gastrointestinal side effects and increased patient compliance due to more convenient dosing regimens. Its main indications for use include treatment of mild to moderate infections of the upper and lower respiratory tract, [1] as well as skin infections [3]. Azithromycin is also highly effective against urogenital infections such as *Chlamydia *[4] and *gonorrhea *[5] when administered as a single one-gram dose. As well, its anti-inflammatory role in cystic fibrosis was also established [6]. Recently, a large study examining treatment of trachomas(almost 1000 people) was published, that documented that the prevalence and intensity of infection fell dramatically and remained low for two years after one treatment[7].

Animal reproductive studies found no evidence of impaired fertility or harm to the fetus following azithromycin exposure [8-10]. Only limited information on the effects of azithromycin use during human pregnancy is available. One study suggested that azalide macrolide was associated with limited transplacental transfer as only 2.6% of the drug that reached the maternal arterial side crossed the placenta. [11].

Although several studies are available on the gestational use of azithromycin, the focus of these studies was to establish the efficacy rather than to examine its safety in pregnancy [12-16]. Effectiveness of the drug for numerous indications were well established in these reports, however no information was provided regarding the gestational age at time of exposure or pregnancy outcomes. There was an observational cohort study that described the outcomes of pregnancies in women who were prescribed newly marketed drugs by their general practitioners. There were 11 reported exposures to azithromycin during the first trimester, resulting in one elective abortion, one ectopic pregnancy and 10 healthy, full-term babies[17]. Another study investigating the efficacy of azithromycin in reducing lower genital ureaplasma colonization in 32 women at risk of pre-term delivery found no adverse neonatal outcomes [18]. Finally, two cases of women with scrub typhus treated successfully with three-day courses of azithromycin in the second trimester reported healthy pregnancy outcomes [19].

All of these primary infections that are treated with azithromycin are common among women of child bearing age, and coupled with the fact that at least 50 percent of pregnancies are unplanned, 8 the likelihood of azithromycin being prescribed during pregnancy is very high. Consequently, there is a need for evidence-based data related to the fetal safety of azithromycin. Our primary objective was to ascertain if there is an increased risk of major malformations (above the baseline risk of 1–3%) in women exposed to azithromycin during pregnancy. Secondary outcomes of interest included rates of spontaneous abortions, fetal distress, gestational age at birth and mean birth weight.

## Methods

The Motherisk Program, is a teratogen information service at The Hospital for Sick Children in Toronto, Canada, where women and their health care providers can call to receive information regarding exposures during pregnancy such as drugs, chemicals, radiation and infectious diseases. If a woman was currently pregnant and taking azithromycin, she was asked if she would be interested in enrolling in a study where the outcome of her pregnancy would be followed up.

At the initial interview, a standardized intake form was completed, which recorded details of maternal demographics, medical and obstetrical histories, concurrent drug use, and other exposures. In addition, informed consent was provided by the woman to allow for follow-up following the birth of her baby.

All the women were contacted by the time the infant was a year old. After completion of the standardised questionnaire that was used to record details of the baby's birth and subsequent health, the mothers were requested to give permission for the interviewer to send a letter to the child's physician, to confirm the medical details they had provided, to eliminate any possible bias by only relying on the mother's report.

Women exposed to azithromycin were compared to two comparison groups consisting of women not exposed to azithromycin who were matched by maternal age (± 2 years), gestational age at the time of call (± 2 weeks), alcohol and cigarette use. Comparison group #1 (disease matched) consisted of women with similar infections, who had used other antibiotics, which are considered safe to use during pregnancy (erythromycin, amoxicillin, clarithromycin, clindamycin). The second comparison group consisted of women exposed to non-teratogenic drugs (acetaminophen, and other over-the-counter medications) who did not have an infection. Rates of major malformations and secondary outcomes of interest were compared among the three groups. A major malformation was defined as any anomaly that has an adverse effect on either the function or the social acceptability of the child [20]. Other pregnancy outcomes including spontaneous abortion(SA), therapeutic abortion (TA). Fetal death and other complications were also compared. This protocol was approved by the Research Ethics Board at the Hospital for Sick Children.

Continuous data among patients and control subjects were compared using one-way analysis of variance (ANOVA). The Kruskal Wallice rank sum test was used to compare continuous data that did not follow normal distribution. Categorical data were compared by χ^2 ^analysis or Fisher's exact test, whenever appropriate.

## Results

The outcome of 123 pregnancies with gestational use of azithromycin was ascertained as well as 123 in each of two comparison groups. In the azithromycin group, 88 (72%) exposures occurred during the first trimester, 23 (19%) in the second trimester, and 12 (9%) in the third trimester. Five women used the drug more than once, due to recurrent infections during the pregnancy. The indication for azithromycin use among the exposed group, were for the most part, respiratory infections (82%) including bronchitis, sinusitis and pneumonia, whereas only 18% of the women used azithromycin for genitourinary infections. Treatment for chlamydia was with a single one-gram oral dose of azithromycin, with no further recurrences. The average treatment for URTI's was for 5 days: day one 250 mg 2/d and day 2–5 250 mg 1/d. There were no differences in the maternal characteristics between the study group and the two comparison groups.

The outcomes in the exposed group were 113 live births; 6 spontaneous abortions; 3 fetal deaths and 1 therapeutic abortion (for which no anomalies were detected). There were no significant differences in the rates of major malformations between women exposed to azithromycin (3.4%), and those in the disease-matched and non-teratogen (2.3% and 3.4%, respectively). Table 1

**Table 1:** Pregnancy Outcomes

**Variables**	**Azithromycin**		**Disease-matched**		**Non-teratogens**		**Azithromycin vs. antibiotics**		**Azithromycin vs. Non-teratogens**		**Three groups**	**P-value overall**
	**N**	**%**	**N**	**%**	**N**	**%**	**χ^2 ^(df = 1)**	**P**	**χ^2 ^(df = 1)**	**P**	**χ^2 ^(df = 2)**	**P**

Live Birth	113	91.9	117	95.1	114	92.7	0.01	0.92	0.95	0.33	0.58	0.75
SA	6	4.9	3	2.4	8	6.5	0.92	0.34	0.04	0.85	2.34	0.31
Fetal Distress	19	16.8	24	20.5	26	22.8	5.70	0.02	0.64	0.42	1.72	0.42
Major malformation	3	3.4	2	2.3	3	3.4						0.89
	Mean	SD	Mean	SD	Mean	SD					χ^2 ^(df = 2)	
Gest age @birth (wks)	39	2	39	2	40	1					0.56	0.76
Birth wt (g)	3460	587	3522	491	3546	501					1.29	0.52

The details of the major malformations are documented in Table 2. Of note, the three malformations in the exposed group, were in the children of mothers who were treated with azithromycin for URTI. Statistical analysis of secondary outcomes did not detect differences among the three groups in terms of pregnancy outcome, fetal distress, preterm delivery rates, and mean birth weight. Table 1

**Table 2:** Malformations

AZITHROMYCIN-EXPOSED GROUP	• Sensory neural deafness (profound congenital) • Tracheomalacia, incompetent oesophageal sphincter and gastroesophageal reflux • Congenital diaphragmatic hernia
DISEASE-MATCHED GROUP	• Ductus arteriosus persistence • Hydronephrosis of the left kidney
NON-TERATOGEN GROUP	• VSD • Hypospadias • Diaphragmatic hernia

## Discussion

To our knowledge, this is the first prospective study that evaluated the fetal safety of azithromycin use during pregnancy. We feel that it confirms, as with the macrolides, that this drug appears to be relatively safe for use during pregnancy[21,22].

Single-dose azithromycin therapy presents a distinct advantage over multiple doses of other antibiotics, and it appears to be the most cost-effective agent for many indications [23]. This is particularly true in the case of adolescent patients and asymptomatic infections, where many patients are unlikely to comply with multi-dose therapy [24]. The combination of increased efficacy, increased compliance, and fewer adverse effects than with other macrolide antibiotics, often makes azithromycin a preferred prescribing option for physicians [25]. However, a lack of evidence-based data regarding the safety of the drug often forces health-care providers to face the predicament of estimating the reproductive risks [26] The current recommendations of the Centre for Disease Control in the United States list azithromycin as a drug of choice for the non-pregnant population, but as an alternative therapy for the pregnant patient [27].

Sexually transmitted diseases may be acquired or become apparent during pregnancy, [28] consequently, it is critical that screening and treatment for infections such as syphilis, gonorrhea and chlamydia be conducted to prevent both maternal and fetal morbidity and mortality [29]. Genital infections may remain undiagnosed during pregnancy, as they are often asymptomatic. Subsequently, consequences of treatment failure during pregnancy can be of greater concern as serious sequelae can result, including increased rates of pregnancy loss, low birth weight, preterm birth, neonatal mortality and congenital infections. In the case of chlamydia-infected populations, the benefits of treating the infection, potentially outweigh the possible risks associated with the drug, given that perinatal transmission of the infection can be of great concern.

The main limitation of this study is the small sample size, as it only had an 80% power to detect a 4.5-fold increase in the rate of major malformations with alpha value of 0.05. Approximately 800 cases in each group would be required to detect a two-fold increase in risk of relatively common malformations and thousands would be required to detect rare defects. However, despite the relatively small sample size, we had 2 other groups of women to make comparisons, thereby strengthening the limited power of the study.

Of note, all 15 women in our study who were prescribed azithromycin for chlamydia infections chose to initiate its use after being informed of the possible concerns if the infection was left untreated during their pregnancy. In contrast, most women taking the drug to treat respiratory infections consisted of primarily inadvertent exposures or second-line therapy.

## Conclusion

The results of this prospective study of 123 pregnant women exposed to azithromycin do not suggest that there is a greater risk for major malformations above the baseline rate of 1%–3%. This evidence-based data will be particularly useful for health care providers in their decision-making, regarding the treatment of pregnant women for infections such as chlamydia when azithromycin is considered to be the drug of choice.

## Competing interests

The author(s) declare that they have no competing interests.

## Authors' contributions

***MS ***conceived and designed the study, analysed the data and wrote the manuscript. ***CW ***assisted in designing the study and editing the manuscript. ***GK ***edited the manuscript and assisted in the design of the study. ***AE ***helped design study and write the paper

## Pre-publication history

The pre-publication history for this paper can be accessed here:


